# Search for a Functional Genetic Variant Mimicking the Effect of SGLT2 Inhibitor Treatment

**DOI:** 10.3390/genes12081174

**Published:** 2021-07-29

**Authors:** Siqi Wang, M. Abdullah Said, Hilde E. Groot, Peter J. van der Most, Chris H. L. Thio, Yordi J. van de Vegte, Niek Verweij, Harold Snieder, Pim van der Harst

**Affiliations:** 1 Department of Cardiology, University Medical Center Groningen, University of Groningen, 9700 RB Groningen, The Netherlands; s.wang01@umcg.nl (S.W.); m.a.said@umcg.nl (M.A.S.); h.e.groot@umcg.nl (H.E.G.); y.j.van.de.vegte@umcg.nl (Y.J.v.d.V.); n.verweij@umcg.nl (N.V.); 2 Department of Epidemiology, University Medical Center Groningen, University of Groningen, 9713 GZ Groningen, The Netherlands; p.j.van.der.most@umcg.nl (P.J.v.d.M.); c.h.l.thio@umcg.nl (C.H.L.T.); h.snieder@umcg.nl (H.S.); 3 Department of Cardiology, Division of Heart and Lungs, University Medical Center Utrecht, University of Utrecht, 3584 CX Utrecht, The Netherlands

**Keywords:** SGLT2 inhibitor, heart failure, UK Biobank, genetic variants

## Abstract

SGLT2 inhibitors (SGLT2i) block renal glucose reabsorption. Due to the unexpected beneficial observations in type 2 diabetic patients potentially related to increased natriuresis, SGLT2i are also studied for heart failure treatment. This study aimed to identify genetic variants mimicking SGLT2i to further our understanding of the potential underlying biological mechanisms. Using the UK Biobank resource, we identified 264 SNPs located in the *SLC5A2* gene or within 25kb of the 5′ and 3′ flanking regions, of which 91 had minor allele frequencies >1%. Twenty-seven SNPs were associated with glycated hemoglobin (HbA1c) after Bonferroni correction in participants without diabetes, while none of the SNPs were associated with sodium excretion. We investigated whether these variants had a directionally consistent effect on sodium excretion, HbA1c levels, and *SLC5A2* expression. None of the variants met these criteria. Likewise, we identified no common missense variants, and although four SNPs could be defined as 5′ or 3′ prime untranslated region variants of which rs45612043 was predicted to be deleterious, these SNPs were not annotated to *SLC5A2*. In conclusion, no genetic variant was found mimicking SGLT2i based on their location near *SLC5A2* and their association with sodium excretion or HbA1c and *SLC5A2* expression or function.

## 1. Introduction

Sodium-glucose co-transporter-2 (SGLT2) is the primary transporter in the proximal tubule of the kidney [1] and reabsorbs over 90% of the glucose from the glomerular filtrate [2]. SGLT2 is encoded by the *SLC5A2* gene, which is located on chromosome 16 (*16p11.2*) [3].

SGLT2 inhibitors (SGLT2i) block renal glucose reabsorption, resulting in increased urinary glucose excretion, but also between a 30% and 60% increase in urinary sodium excretion [4], and blood glucose reduction [5]. Originally used for the treatment of type 2 diabetes (T2D), SGLT2i was the first anti-diabetic drug shown to reduce the risk of hospitalization for heart failure (HF) in these patients [6]. HF is a complex clinical syndrome in which the heart is unable to pump a sufficient amount of blood for the body’s requirements and is caused by a structural or functional impairment of the contractility or filling of the ventricles. HF is a major cause of cardiovascular morbidity and mortality worldwide, leading to a heavy economic burden on society [7]. A recent study reported that the use of SGLT2i was associated with a lower risk of worsening HF or death from a cardiovascular cause among patients with HF with a reduced ejection fraction, regardless of their diabetic status [8]. SGLT2i is relatively new as a treatment for HF, and little is known of its mechanism of action on HF or its possible side effects. Therefore, finding genetic variants that mimic SGLT2i may contribute to understanding the mechanisms underlying HF treatment with SGLT2i.

**Figure 1:**
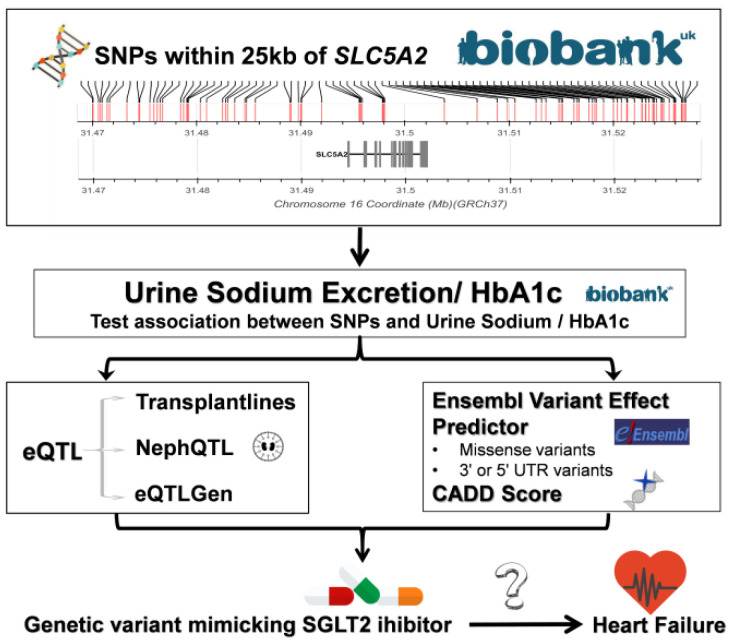
Study design.

This study aimed to identify genetic variants which mimic SGLT2i and use these to investigate the possible causal links with HF and other biomarkers and diseases to scan for potential side effects (Figure 1).

## 2. Materials and Methods

### 2.1. UK Biobank Study Population 

The UK Biobank population and study design have been described in detail previously [9]. Briefly, the UK Biobank is a large prospective cohort study, including more than 500,000 participants aged between 40–69 years old that were included between 2006 and 2010 [10]. All participants provided informed consent.

### 2.2. Genotyping and Imputation in the UK Biobank 

The genotyping and imputation procedures in the UK Biobank have been described in detail previously [11]. Briefly, participants were genotyped using either the custom UK Biobank Axiom^TM^ or UK Biobank Lung Exome Variant Evaluation (UK BiLEVE) Axiom^TM^ from Affymetrix. These arrays, respectively, include 820,967 and 807,411 single nucleotide polymorphisms (SNPs), insertion and deletion markers with >95% shared contents [11]. Genotyping, quality control before imputation, and imputation based on merged UK10K and 1000 Genomes phase 3 panels were performed by the Wellcome Trust Center for Human Genetics.

### 2.3. Candidate SNP Selection

To identify genetic variants mimicking SGLT2i, we applied several methods. First, we selected variants in or near the *SLC5A2* gene in the UK Biobank and tested their associations with the urinary sodium/creatinine ratio (UNa/Cr) and predicted 24 h urinary sodium excretion in all individuals, and with glycated hemoglobin (HbA1c) in participants without diabetes. Diabetes was defined by having type I, type II, or gestational diabetes or taking anti-diabetic drugs at the time of inclusion in the UK Biobank. The effects of variants on gene expression were obtained from the TransplantLines cohort, NephQTL, and eQTLGen data resources. Independent variants with directionally consistent effects on *SLC5A2* gene expression and UNa/Cr, 24 h urinary sodium excretion, or HbA1c, were considered SGLT2i-mimicking genetic variants. That is, allelic variants associated with lower *SLC5A2* gene expression were expected to lead to less effective sodium or glucose reabsorption resulting in higher urinary sodium or lower HbA1c levels and vice versa. Finally, we assessed the functional impact of the genetic variants through the Ensembl Variant Effect Predictor (VEP) tool (human species, GRCh37.p13)and the Combined Annotation Dependent Depletion (CADD) tool (GRCh37-v1.6).

### 2.4. SGLT2i Variants Based on Position

Using the UK Biobank resource, we extracted all of the genetic variants with minor allele frequencies (MAFs) ≥ 0.01 from the *SLC5A2* gene locus and 25 kb of the 5′ and 3′ flanking region (chromosome 16, hg19 positions 31,469,439-31,527,091). Since HbA1c values reflect long-term glycemic status over a period of two to three months [12], and beneficial effects of SGLT2i may be related to increased natriuresis, these genetic variants were then tested against HbA1c (mmol/mol), UNa/Cr (mg/g), and predicted 24 h urinary sodium excretion (mg/day) in the UK Biobank using linear regression models, including values within mean ± 5SD separately. The concentration of urinary sodium was obtained from a random spot urine sample, details about measurements could be found elsewhere [13]. Considering the effects of urinary dilution [14], the UNa/Cr was used to minimize the inaccuracy associated with spot urine collections. We also use predicted 24 h urinary sodium excretion that was estimated from age, weight, height, and the concentration values of urinary sodium and creatinine by using the sex-specific [15] Kawasaki equation [16]. Variants were considered more likely to be potential SGLT2i variants if they were associated with a change in sodium and/or HbA1c and therefore prioritized as candidates for genetic variants. HbA1c was measured by High-Performance Liquid Chromatography analysis on a Bio-Rad Variant II Turbo analyzer [17].

### 2.5. SLC5A2 eQTL Analyses

The TransplantLines [18] cohort and the online NephQTL [19] and eQTLGen [20] data resources were used to explore the effect of the genetic variants on *SLC5A2* gene expression.

TransplantLines includes kidney samples of 188 European donors and is potentially a viable resource, considering *SLC5A2* is almost exclusively expressed in kidney tissue [21]. TransplantLines is a prospective cohort study of organ donors and recipients, including all different types of solid organ transplant recipients and organ donors [18]. Kidney samples were taken from living donors (*n* = 35), donated after brain death (*n* = 104) or non-heart-beating death (*n* = 49). Written informed consent was obtained from all living donors prior to inclusion, and the TransplantLines study protocol was approved by the Institutional Research Board of the University Medical Center Groningen (METc 2014/077). eQTL analysis was performed as follows: Samples were genotyped on the Illumina CytoSNP 12 v2 array [22] and imputed on the Michigan Server [23] to the Haplotype Reference Consortium [24] dataset. Whole-genome gene expression was assayed by Illumina HumanHT-12 v4 Expression BeadChips [25]. Expression and genotype data were available for 328 biopsies of healthy kidneys obtained from 188 donors, the analyses have been described elsewhere [22,26].

We used data from the NephQTL resource as a second independent eQTL resource to identify other variants that explain gene expression variance of *SLC5A2* or to validate genetic variants from TransplantLines. The NephQTL resource is a database of cis-eQTLs of the glomerular and tubulointerstitial tissues. Kidney samples were obtained from 187 participants in the Nephrotic Syndrome Study Network (NEPTUNE), a prospective and longitudinal cohort [19]. We used the data generated from tubulointerstitial tissues, which included 166 participants, as *SLC5A2* is mainly expressed on the apical membrane of the epithelial cells of the proximal tubule [27]. *SLC5A2* gene expression in various tissues are shown in Supplementary Figure S1. Information regarding the population and methods in NephQTL have been described in detail previously [19], in which cis-eQTL analysis was performed by using MatrixEQTL [19], and Affymetrix 2.1 ST chips [28] were used to generate gene expression data from microdissected tubulointerstitial tissues. Informed consent was acquired from all of the participants, and all procedures were performed according to the ethical standards of the institutional review boards overseeing the NEPTUNE study [19].

Finally, as a third independent resource, we used expression data from the eQTLGen database. The eQTLGen database includes data on 19,960 genes expressed on autosomal chromosomes in 31,684 blood samples from 37 cohorts [20]. The levels of gene expression were profiled by Illumina, Affymetrix U291, Affymetrix HuEx v1.0 ST expression arrays, and by RNA-seq [20]. More information about the eQTLGen resource can be found in more detail elsewhere [20]. We used the cis-eQTL data from eQTLGen to check the effect of SNPs found in the UK Biobank on *SLC5A2* gene expression in blood.

### 2.6. Functional Impact of SLC5A2 Variants

We used the Ensembl [29] VEP tool to assess the functional impact of variants selected from the UK Biobank. More details about VEP can be found elsewhere [30]. The Combined Annotation Dependent Depletion (CADD) tool (GRCh37-v1.6) is one of the most widely used tools for predicting the deleteriousness of human genetic variation, and a CADD PHRED-scaled score of an SNP greater than 12.37 was considered pathogenic [31].

### 2.7. Exclusion Criteria

**Figure 2:**
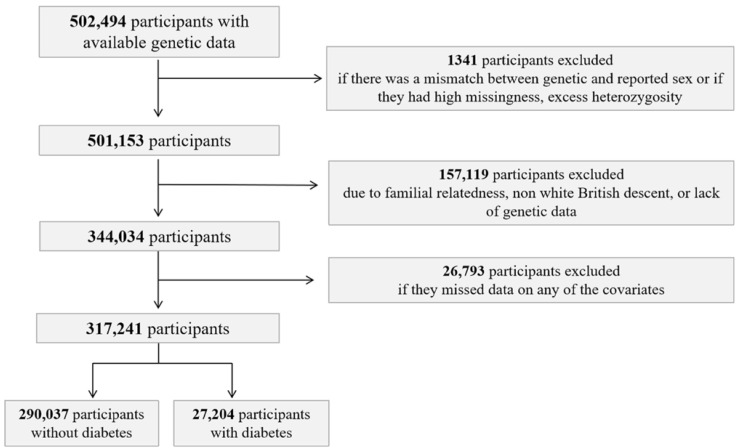
Flowchart of study population selection from the UK Biobank. It depicts the sample selection in the UK Biobank study. Diabetes was defined by having type I, type II, gestational diabetes, or taking anti-diabetic drugs at time of inclusion in the UK Biobank.

Participants were excluded if there was a mismatch between genetic and reported sex or if they had high missingness, excess heterozygosity (*n* = 1341). Participants were furthermore excluded due to familial relatedness, nonwhite British descent, lack of genetic data (*n* = 157,119), or missed data on any of the covariates (*n* = 26,793) (Figure 2).

### 2.8. Statistical Analysis

Linear regression analyses were performed to investigate the association between the genetic variants and UNa/Cr, predicted 24 h urinary sodium excretion, or HbA1c. If a genetic variant was found to mimic SGLT2i, linear regression analyses were performed to investigate the association between the genetic variant and the related continuous traits of cardiovascular diseases (CVDs), and logistic regression analyses were performed to study the association between the genetic variant and HF in the UK Biobank. All regression analyses were adjusted for age at inclusion, sex, genotyping chip, and the first 30 genetic principal components. All regression analyses were performed using STATA version 16. A regional association plot between the tested SNPs and HbA1c was created using LocusZoom [32]. We set the two-sided α at 0.05 and applied Bonferroni correction to account for multiple testing.

## 3. Results

### 3.1. Population Characteristics

A total of 317,241 individuals of European ancestry participating in the UK Biobank were included in this study (Figure 2). Of the included participants, 53.49% (*n* = 169,688) were female and the average age (SD) was 57.39 (8.0) years at the initial assessment visit. Detailed baseline characteristics for the UK Biobank participants are shown in Table 1.

### 3.2. SLC5A2 Variants in UK Biobank

**Figure 3:**
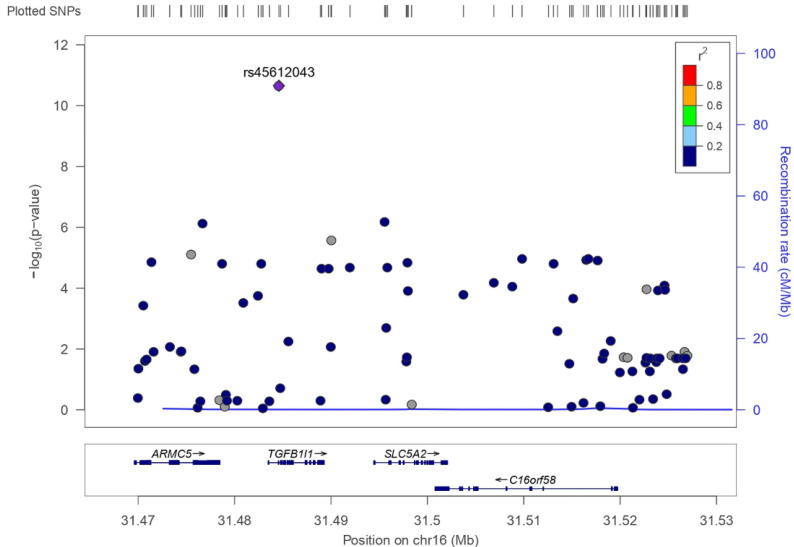
The association of SNPs within 25 kb of the *SLC5A2* gene with HbA1c. Regional plots for HbA1c-associated SNPs located in the *SLC5A2* gene or within 25 kb of the 5′ and 3′ flanking regions in the UK Biobank. Linkage disequilibrium (r^2^) is shown between rs45612043 (purple diamond) and the other SNPs in this plot. Unknown linkage disequilibrium estimates are shown in gray color.

A total of 264 genetic variants available in the UK Biobank were located in the *SLC5A2* gene or within 25 kb of the 5′ and 3′ flanking regions. A total of 91 SNPs met the MAF threshold of >1% (Supplementary Table S1). These 91 SNPs were first tested against UNa/Cr and predicted 24 h urinary sodium excretion, 43 of them associated with UNa/Cr (*p* < 0.05, *n* = 316,923, Supplementary Table S2) and 44 with predicted 24 h urinary sodium excretion (*p* < 0.05, *n* = 317,241, Supplementary Table S3), but none of them remained significant after Bonferroni correction (*p* < [0.05/(91 [number of SNPs] × 3 [number of traits])] = 1.83 × 10^−4^). Then we tested for HbA1c, and Figure 3 depicts the association of these 91 SNPs with HbA1c. A total of 27 SNPs were significantly associated with HbA1c after Bonferroni correction (*p* < [0.05/(91 × 3) = ] 1.83 × 10^−^^4^, *n* = 289,803) in non-diabetic individuals (Table 2), and linkage disequilibrium between the variants is shown in Supplementary Figure S2. The strongest variant, rs45612043, was genome-wide significant (MAF = 0.043, *p* = 2.22 × 10^−^^11^). We took forward these 27 genetic variants as potential candidates for genetic variants to mimic SGLT2i and performed lookups in three eQTL resources to investigate if they truly explain variance in gene expression values of the *SLC5A2* gene with directionally consistent effects on both HbA1c and *SLC5A2* gene expression.

### 3.3. Effects on Gene Expression

**Figure 4:**
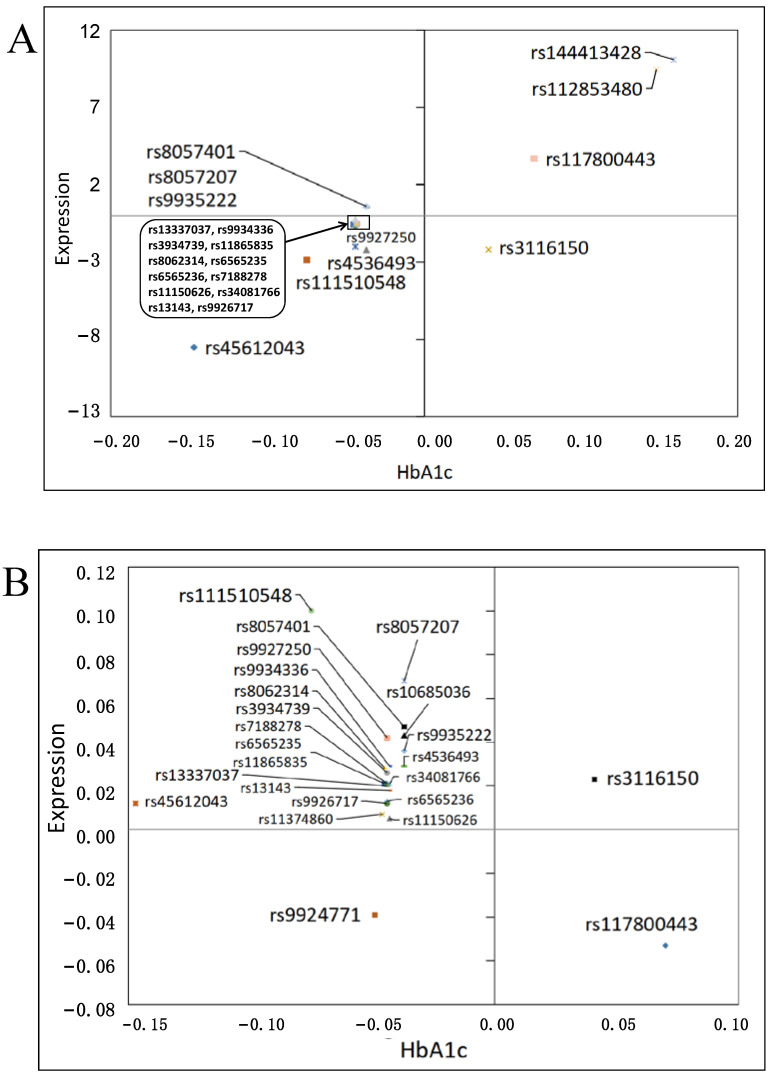

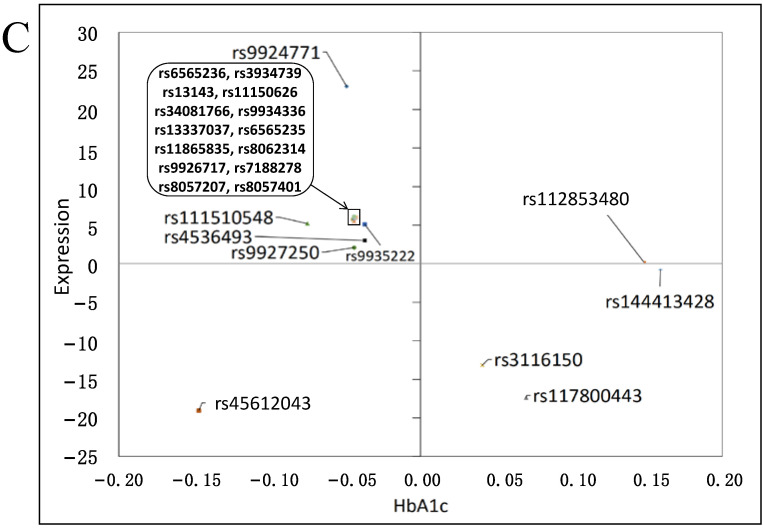
Effects of SNPs on HbA1c and *SLC5A2* gene expression. Schemes follow another format. X-axis: Betas for HbA1c (mmol/mol) from the UK Biobank. Y-axis: (**A**) Estimates for *SLC5A2* gene expression in the TransplantLines cohort, (**B**) Betas for *SLC5A2* gene expression in the NephQTL resource, (**C**) Z-scores for *SLC5A2* gene expression in the eQTLGen consortium.

Three eQTL resources were queried to identify SNPs associated with *SLC5A2* gene expression. The first resource was the TransplantLines study, which included data on 188 European kidney donors, with a mean (SD) age of 46.4 (14.6) years. Over half (*n* = 98, 52.13%) of the donors were female. A total of 23 out of the 27 SNPs which were associated with HbA1c after Bonferroni correction in the UK Biobank could be found in the TransplantLines resource. Of these, 19 SNPs had a directionally consistent effect on both *SLC5A2* gene expression and HbA1c, but the associations with gene expression were not statistically significant (Figure 4A, Supplementary Table S4).

The NephQTL resource included 166 subjects with tubulointerstitial data, with a median (interquartile range) age of 36 (17–56) years and less than half (*n* = 51, 30%) of the subjects were female. In this resource, 24 of the 27 candidate SNPs could be found. Two SNPs, rs3116150 and rs9924771, had directionally consistent effects on both *SLC5A2* gene expression and HbA1c, but effects on gene expression did not reach statistical significance (Figure 4B, Supplementary Table S5).

In the eQTLGen consortium, which consists of 31,684 blood samples from 37 datasets that were all preprocessed and analyzed in a standardized way, a total of 24 out of the 27 candidate genetic variants were available. Of these, only rs45612043 and rs112853480 were associated with a directionally consistent lower effect on HbA1c and gene expression. Their association with gene expression was, however, not statistically significant (Figure 4C, Supplementary Table S6).

### 3.4. Functional Impact

The 27 potential genetic variant candidates were subsequently examined using the VEP tool to identify protein-coding variants. None of the genetic variants were annotated as a missense variant. We did find four 5′ or 3′ prime untranslated region (UTR) variants (Table 3) of which one (rs45612043) was predicted to be deleterious by the CADD PHRED scaled score, but these were annotated to the *ARMC5* or *TGFB1I1* genes and not to *SLC5A2*. The CADD score for all SNPs significantly associated with HbA1c in UK Biobank is shown in Supplementary Table S7.

## 4. Discussion

SGLT2i have been demonstrated to reduce cardiovascular events in HF patients, especially hospitalization for HF, compared to placebos [33]. The results of a recent large clinical trial (*n* = 3730) showed that SGLT2i was associated with a significantly lowered risk of HF hospitalization and cardiovascular death (hazard ratio = 0.75 (95% CI 0.65–0.86); *p* < 0.001) in HF patients with or without diabetes [34]. In addition, SGLT2i was associated with a slower annual decline rate of the estimated glomerular filtration rate (−0.55 vs. −2.28 mL per minute per 1.73 m^2^ of body surface area per year, *p* < 0.001), accompanied by a lower risk of serious renal outcomes [35]. The underlying mechanisms of SGLT2i treatment for HF possibly include decreased blood pressure, inflammation, increased cardiac energy metabolism, erythropoiesis, and adverse cardiac remodeling [36]. However, this is still a point of debate. In this study, we aimed to identify genetic variants mimicking SGLT2i to allow the investigation of the potential mechanisms underlying the effect of SGLT2i on HF and to test for potential side effects of SGLT2i. We evaluated 91 SNPs within 25kb of the *SLC5A2* gene region that were available in the UK Biobank dataset and estimated the strength of their association with sodium or HbA1c to prioritize them as candidates for genetic variants by strengthening the biological plausibility for the potential SGLT2i variants [37]. Although glycaemic control is reported to be unlikely to be related to the benefits of SGLT2i on CVD [38], decreasing blood glucose is still the main characteristic of SGLT2i. This may have been less evident in our population, however, as the mean (SD) HbA1c value in the present study was 35.93 (6.45) (mmol/mol), while none of the SGLT2i clinical trials with CVD outcomes recruited patients with HbA1c < 48 mmol/mol (< 6.5%) [39].

Previous studies suggested *SLC5A2* genetic variants were associated with urinary glucose excretion [40], glucose homeostasis [41], and diabetes [42]. One study (*n* = 2229) selected six common SNPs with MAFs ≥0.05 to cover the *SLC5A2* gene region and 2 kb of the 3′ flanking regions based on 1000 Genomes Project data. Of the six SNPs, rs11646054 was excluded as it resisted multiplex assay design for MassARRAY and TaqMan assay design for allelic discrimination. Rs3116149 was excluded because it was monomorphic in all patients, leaving rs9934336, rs9924771, rs3813008, and rs3116150 for further analyses. The association between these four variants with HbA1c was assessed but yielded no significant associations [43]. Three of the SNPs (rs9934336, rs3813008, rs3116150) were also selected by a study with 1684 individuals to cover all variants with MAFs ≥ 0.05 and pairwise r^2^ ≥ 0.8 within the *SLC5A2* gene, 2 kb of the 5′ flanking region, and 1 kb of the 3′ flanking region [42]. Unlike the first study, they found one SNP, rs9934336, that was associated with HbA1c (*p* = 0.023). These three SNPs were also later tested in a study with 907 individuals [41], where rs9934336 was again found to be nominally associated with several glycaemic markers (*p* < 0.05). Whether HbA1c was among the tested glycaemic markers was, however, not reported. The HbA1c lowering effect of the A allele of rs9934336 was confirmed in our study (β = −0.043 [SE 0.010]; *p* = 2.11 × 10^−^^5^). Unlike the previous studies, we found rs3116150 was associated with changes in HbA1c, also after Bonferroni correction. Rs11646054, rs3116149, and rs3018008 were also tested but were not associated with HbA1c in the UK Biobank. Finally, a recent genome-wide association study reported the association between rs13337037 and glycosuria (OR per effect allele = 1.42 (95% CI 1.30–1.56); *p* = 1.97 × 10^−13^) [44]. Although one of the main effects of SGLT2i is an increased urinary glucose excretion [37], rs13337037, which was also identified in proximity to *SLC5A2* and associated with HbA1c in the current study, did not meet further criteria to be considered a potential SGLT2i variant.

Compared to previous studies, we explored a larger genetic region for a possible SGLT2i-mimicking genetic variant and adopted a more lenient MAF threshold, considering the large sample size of the UK Biobank. We adopted stringent criteria to prioritize genetic variants as valid instrumental variables by testing for a directionally consistent effect on HbA1c and *SLC5A2* gene expression. Despite querying three independent eQTL data repositories, the VEP tool as well as the CADD tool to identify variants with functional effects, we found no variants fulfilling these criteria. Only rs45612043, the SNP with the strongest association with HbA1c in our study (*p* = 2.22 × 10^−11^), was predicted to be deleterious by the CADD PHRED scaled score, but it was annotated to *TGFB1I1* which is involved in cell proliferation [45].

Since previous studies exploring the effect of the genetic variants in the *SLC5A2* gene on protein function or gene expression are lacking [43], no data from other literature on the association of the SNPs with *SLC5A2* are available. Larger and more detailed studies on kidney tissue expression of *SLC5A2* could facilitate the discovery of variants that biologically mimic SGLT2i. This will enable future studies to use these genetic variants as instrumental variables in Mendelian randomization studies and thereby provide potentially novel biological insights into the mechanisms underlying SGLT2i.

The major strengths of this study are the double-positive control for selecting potential instrumental variables for SGLT2i, which consisted of testing concordant directional effects of the SNPs on *SLC5A2* gene expression and UNa/Cr, predicted urinary sodium excretion or HbA1c in more than 290,000 individuals, as well as the use of multiple resources to investigate the biological consequences of the genetic variants. The 27 SNPs that were significantly associated with HbA1c in this large cohort of non-diabetic individuals might be interesting variants to follow up in future pharmacogenomics studies investigating their effects on the efficacy of SGLT2i in HF treatment. There are also limitations. Firstly, the spot urine sample was collected at the end of a 2-h visit [46] rather than in the morning, and the participant’s diet before urine collection was not recorded by the UK Biobank. Furthermore, 24 h urinary sodium excretion was estimated based on the spot urine sample rather than collecting 24 h urine samples. Second, although all associations were tested in individuals without diabetes in order to exclude potential effects induced by glycaemic dysregulation or anti-diabetic drugs, there may be individuals with undiagnosed diabetes or individuals who are treated and diagnosed in outpatient settings but did not report this at visits to the UK Biobank assessment centre. This could introduce some ascertainment bias, but such classification errors are likely biased towards the null and would rather underestimate than overestimate the observed effects. The third limitation of our study is that, ideally, given the function of SGLT2, our analyses should have been performed on urinary glucose levels or excretion rates but these phenotypes were not measured in the UK Biobank. Instead, we used HbA1c levels as a surrogate, but the range of HbA1c values in the UK Biobank is largely within the normal range potentially limiting the sensitivity of our search for a functional variant mimicking the effect of SGLT2 inhibitor treatment. The fourth limitation is that the analyses of these genetic variants tested against HbA1c were performed only in individuals of white British descent, which may limit its generalizability to other racial or ethnic groups. Finally, the limited sample size of the kidney eQTL datasets we used in this study may have limited the power to detect associations. The analyses could be repeated when larger eQTL data resources are available.

## 5. Conclusions

In conclusion, we performed a large-scale search within 25kb of the *SLC5A2* gene locus, but did not identify a genetic variant that could be used as an SGLT2i-mimicking genetic variant based on their association with UNa/Cr, predicted 24 h urinary sodium excretion, or HbA1c, and their association with *SLC5A2* gene expression or impact on protein function.

## Figures and Tables

**Table 1:** Baseline Characteristics of All Included UK Biobank Participants.

Characteristics	Mean (SD)/*n* (%)
Age (years)	57.39 (7.99)
Female	169,688 (53.49%)
BMI (kg/m^2^)	27.39 (4.73)
Diastolic blood pressure (mm Hg)	82.15 (8.54) *
Systolic blood pressure (mm Hg)	133.75 (17.95) *
Resting heart rate	69.32 (11.24) **
HbA1c (mmol/mol)	35.93 (6.45)
LDL (mmol/L)	3.57 (0.87) #
HDL (mmol/L)	1.45 (0.38) ##
Urinary sodium/creatinine ratio (mg/g)	2145.87 (1198.64)
Predicted 24 h urinary sodium excretion (mg/day)	1894.40 (563.79)
Heart failure	9850 (3.1%)
Hypertension	117,196 (36.97%) *
Diabetes	27,204 (8.58%)

Continuous variables are presented as mean ± SD and binary variables as percentages, BMI = Body mass index, SD = Standard Deviation, LDL = Low density lipoprotein, HDL = High density lipoprotein. * 237 of 317,241 participants missed data on blood pressure. ** 186 of 317,241 participants missed data on resting heart rate. # 14,699 of 317,241 participants missed data on LDL, ## 39,943 of 317,241 participants missed data on HDL.

**Table 2:** SNPs Significantly Associated with HbA1c in UK Biobank.

SNP	CHR	EFAL	NEFAL	MAF (Minor Allele)	β	SE	*p*
rs45612043	16	C	A	0.043 (C)	−0.147	0.022	2.22 × 10^−11^
rs9924771	16	G	A	0.348 (A)	−0.049	0.010	6.53 × 10^−7^
rs111510548	16	C	T	0.098 (C)	−0.075	0.015	7.58 × 10^−7^
rs1251169601	16	C	CAAAAAAAAAAAA	0.410 (C)	0.046	0.010	2.72 × 10^−6^
rs11374860	16	TG	T	0.279 (TG)	−0.046	0.010	7.80 × 10^−6^
rs11865835	16	C	T	0.290 (C)	−0.045	0.010	1.09 × 10^−5^
rs8062314	16	A	C	0.290 (A)	−0.045	0.010	1.11 × 10^−5^
rs6565235	16	T	C	0.290 (T)	−0.045	0.010	1.20 × 10^−5^
rs6565236	16	T	A	0.278 (T)	−0.044	0.010	1.23 × 10^−5^
rs9926717	16	G	A	0.292 (G)	−0.044	0.010	1.40 × 10^−5^
rs144413428	16	A	G	0.015 (A)	0.159	0.037	1.47 × 10^−5^
rs13337037	16	A	G	0.275 (A)	−0.044	0.010	1.56 × 10^−5^
rs7188278	16	T	C	0.290 (T)	−0.044	0.010	1.56 × 10^−5^
rs3934739	16	T	C	0.277 (T)	−0.044	0.010	1.56 × 10^−5^
rs11150626	16	C	T	0.278 (C)	−0.043	0.010	2.06 × 10^−5^
rs9934336	16	A	G	0.277 (A)	−0.043	0.010	2.11 × 10^−5^
rs34081766	16	A	C	0.277 (A)	−0.043	0.010	2.27 × 10^−5^
rs13143	16	T	C	0.278 (T)	−0.043	0.010	2.27 × 10^−5^
rs9927250	16	G	A	0.220 (A)	−0.044	0.011	6.61 × 10^−5^
rs8057207	16	T	C	0.357 (T)	−0.037	0.010	8.26 × 10^−5^
rs112853480	16	C	T	0.016 (C)	0.148	0.038	8.71 × 10^−5^
rs10685036	16	TTA	T	0.359 (TTA)	−0.037	0.010	1.10 × 10^−4^
rs8057401	16	T	C	0.356 (T)	−0.037	0.010	1.14 × 10^−4^
rs9935222	16	A	C	0.354 (A)	−0.037	0.010	1.19 × 10^−4^
rs3116150	16	A	G	0.235 (A)	0.041	0.011	1.24 × 10^−4^
rs4536493	16	G	A	0.312 (A)	−0.037	0.010	1.65 × 10^−4^
rs117800443	16	A	G	0.066 (A)	0.070	0.019	1.80 × 10^−4^

Abbreviations: CHR = Chromosome, EFAL = Effect allele, NEFAL = Non-effect allele, MAF = Minor allele frequency, SE = standard error.

**Table 3:** 3′ and 5′ Prime UTR Variants within 25kb of *SLC5A2* Gene Region.

SNP	CHR	Locus	Allele	Consequence	Symbol	CADD Score
rs9926717	16	31,471,378	G	3′ prime UTR variant, NMD transcript variant	*ARMC5*	8.528
rs111510548	16	31,476,695	C	3′ prime UTR variant	*ARMC5*	4.538
rs45612043	16	31,484,598	C	5′ prime UTR variant	*TGFB1I1*	12.790 *
rs13143	16	31,489,033	T	3′ prime UTR variant, NMD transcript variant	*TGFB1I1*	6.091

Abbreviations: CHR = Chromosome, UTR= Untranslated region, NMD= Nonsense-mediated mRNA decay, * predicted to be pathogenic if higher than 12.37.

## Data Availability

The datasets analysed during the current study are available from the corresponding author upon reasonable request.

## Supplementary Materials

The following are available online at https://www.mdpi.com/article/10.3390/genes12081174/s1. Figure S1: *SLC5A2* gene expression in various tissues, Figure S2: Genetic correlation of the 25 SNPs which significantly associated with HbA1c in UK Biobank, Table S1: SNPs located in the *SLC5A2* gene or within 25 kb of the 5′ and 3′ flanking regions (only SNPs with MAFs > 1% were included), Table S2: The association between SNPs and urinary sodium/creatinine ratio (mg/g) in the UK Biobank, Table S3: The association between SNPs and predicted 24 h urinary sodium excretion (mg/day) in the UK Biobank, Table S4: Effects of SNPs on HbA1c and *SLC5A2* gene expression in the TransplantLines cohort, Table S5: Effects of SNPs on HbA1c and *SLC5A2* gene expression in the NephQTL resource, Table S6: Effects of SNPs on HbA1c and *SLC5A2* gene expression in the eQTLGen consortium, Table S7: CADD score for SNPs significantly associated with HbA1c in UK Biobank.
