# Supplementary material for: Search for a Functional Genetic Variant Mimicking the Effect of SGLT2 Inhibitor Treatment
Source: Genes (Basel). 2021 Jul 29;12(8):1174. doi: 10.3390/genes12081174 (PMC8391850; doi:10.3390/genes12081174)
Supplement: Supplementary file 1 [file genes-12-01174-s001.zip › genes-1308573-supplementary.pdf]

Supplementary Information

Siqi Wang [1,†], M. Abdullah Said [1,†], Hilde E. Groot [1], Peter J. van der Most [2], Chris H. L. Thio [2], Yordi J. van de Vegte [1], Niek Verweij [1], Harold Snieder [2] and Pim van der Harst [1,3,*]

Table S1. SNPs located in the *SLC5A2* gene or within 25kb of the 5' and 3' flanking regions. Only SNPs with MAFs>1% were included.

SNPs	Chr	Locus	Minor allele	Major allele	MAF
rs117947985	16	31,469,967	C	G	0.011
rs117600469	16	31,470,023	G	C	0.015
rs3813002	16	31,470,540	T	C	0.305
rs76210462	16	31,470,729	C	A	0.011
rs151069962	16	31,470,886	A	T	0.064
rs9926717	16	31,471,378	G	A	0.292
rs4889660	16	31,471,604	T	C	0.150
rs35923277	16	31,473,275	G	A	0.045
rs4262961	16	31,474,418	A	G	0.150
rs4362405	16	31,474,505	T	C	0.150
rs11374860	16	31,475,477	TG	T	0.279
rs142376949	16	31,475,864	T	C	0.022
rs55800131	16	31,476,186	G	C	0.093
rs11150624	16	31,476,458	T	C	0.402
rs111510548	16	31,476,695	C	T	0.098
rs142544346	16	31,478,434	TGCCTG	T	0.051
rs13337037	16	31,478,711	A	G	0.275
16:31479023_CT_C	16	31,479,023	C	CT	0.492
rs117392918	16	31,479,094	A	G	0.012
rs4889661	16	31,479,200	T	C	0.402
rs4313820	16	31,480,318	G	C	0.404
rs11150625	16	31,480,922	T	C	0.304
rs117800443	16	31,482,455	A	G	0.066
rs7188278	16	31,482,749	T	C	0.290
rs146241762	16	31,482,942	T	C	0.012
rs11646911	16	31,483,622	T	C	0.402
rs45612043	16	31,484,598	C	A	0.043
rs141227787	16	31,484,758	A	G	0.012
rs117552655	16	31,485,605	T	A	0.023
rs10596	16	31,488,916	G	C	0.402
rs13143	16	31,489,033	T	C	0.278
rs34081766	16	31,489,741	A	C	0.277
rs111332953	16	31,489,994	T	C	0.045
rs1251169601	16	31,490,046	C	CAAAAAAAAAAAA	0.410
rs11150626	16	31,491,982	C	T	0.278
rs9924771	16	31,495,577	A	G	0.348
rs11646054	16	31,495,671	C	G	0.402
rs3116149	16	31,495,726	A	G	0.058
rs9934336	16	31,495,873	A	G	0.277
rs3813007	16	31,497,808	T	A	0.013

rs3813008	16	31,497,881	A	G	0.154
rs144413428	16	31,497,920	A	G	0.015
rs3116150	16	31,498,021	A	G	0.235
rs71375930	16	31,498,364	TCAAAAA	T	0.399
rs4536493	16	31,503,751	A	G	0.312
rs9927250	16	31,506,900	A	G	0.220
rs112853480	16	31,508,833	C	T	0.016
rs11865835	16	31,509,816	C	T	0.290
rs568998581	16	31,510,543	G	T	0.106
rs79414612	16	31,512,564	A	C	0.063
rs3934739	16	31,513,086	T	C	0.277
rs62053177	16	31,513,509	T	C	0.043
rs117803319	16	31,514,762	A	G	0.011
rs79371355	16	31,514,980	C	A	0.063
rs6565234	16	31,515,110	T	C	0.016
rs4889664	16	31,516,210	T	C	0.395
rs6565235	16	31,516,513	T	C	0.290
rs8062314	16	31,516,690	A	C	0.290
rs6565236	16	31,517,648	T	A	0.278
rs4889665	16	31,517,978	T	C	0.089
rs8047112	16	31,518,206	T	G	0.147
rs6565237	16	31,518,348	T	C	0.465
rs11861164	16	31,519,048	A	G	0.096
rs11866003	16	31,519,995	T	G	0.171
16:31520375_GT_G	16	31,520,375	GT	G	0.470
rs35783178	16	31,520,759	C	CT	0.480
rs4889545	16	31,521,276	T	C	0.174
rs4889546	16	31,521,334	A	G	0.099
rs35884660	16	31,522,008	G	C	0.371
rs72790527	16	31,522,649	T	G	0.162
rs7192001	16	31,522,723	T	C	0.470
rs10685036	16	31,522,753	TTA	T	0.359
rs58729087	16	31,523,096	C	T	0.173
rs7187900	16	31,523,113	A	G	0.469
rs4889666	16	31,523,412	C	A	0.371
rs17709110	16	31,523,775	T	C	0.012
rs8050548	16	31,523,801	G	A	0.469
rs9935222	16	31,523,926	A	C	0.354
rs8057326	16	31,524,123	T	C	0.470
rs8057207	16	31,524,580	T	C	0.357
rs8057401	16	31,524,685	T	C	0.356
rs72790535	16	31,524,824	A	G	0.050
16:31525353_CCCTCCCTT_C	16	31,525,353	CCCTCCCTT	C	0.469
rs8062220	16	31,525,774	G	T	0.469
rs8064191	16	31,525,871	A	G	0.469
rs8062603	16	31,525,960	G	A	0.469
rs9935908	16	31,526,534	T	C	0.469
rs56060924	16	31,526,547	G	A	0.165
16:31526674_CA_C	16	31,526,674	CA	C	0.465

rs9922901	16	31,526,823	G	A	0.469
rs111306768	16	31,526,948	C	CATGAAAGAAT	0.475

Abbreviations: Chr=Chromosome, MAF=Minor allele frequency.

Table S2. The association between SNPs and urinary sodium/creatinine ratio (mg/g) in the UK Biobank.

SNP	CHR	Locus	EFAL	NEFAL	Beta	SE	*P*
rs6565235	16	31,516,513	T	C	-9.822	3.309	0.003
rs8062314	16	31,516,690	A	C	-9.820	3.309	0.003
rs7192001	16	31,522,723	C	T	-8.708	2.970	0.003
rs8050548	16	31,523,801	A	G	-8.694	2.968	0.003
rs8057326	16	31,524,123	C	T	-8.694	2.968	0.003
rs7187900	16	31,523,113	G	A	-8.678	2.968	0.003
rs8062220	16	31,525,774	T	G	-8.672	2.968	0.003
rs8064191	16	31,525,871	G	A	-8.670	2.968	0.003
rs8062603	16	31,525,960	A	G	-8.667	2.968	0.003
rs9935908	16	31,526,534	C	T	-8.666	2.968	0.003
rs9922901	16	31,526,823	A	G	-8.658	2.968	0.004
16:31520375_GT_G	16	31,520,375	G	GT	-8.636	2.969	0.004
rs6565237	16	31,518,348	T	C	-8.668	2.984	0.004
rs111306768	16	31,526,948	CATGAA AGAAT	C	-8.659	2.982	0.004
rs11865835	16	31,509,816	C	T	-9.587	3.307	0.004
rs6565236	16	31,517,648	T	A	-9.587	3.310	0.004
16:31525353_CCCTCCCT T_C	16	31,525,353	C	CCCTC CCTT	-8.497	2.972	0.004
16:31526674_CA_C	16	31,526,674	C	CA	-8.490	2.979	0.004
rs35783178	16	31,520,759	CT	C	-8.551	3.001	0.004
rs4889664	16	31,516,210	T	C	8.581	3.040	0.005
rs3934739	16	31,513,086	T	C	-9.333	3.308	0.005
rs7188278	16	31,482,749	T	C	-9.297	3.309	0.005
rs35884660	16	31,522,008	G	C	8.575	3.064	0.005
rs4313820	16	31,480,318	G	C	8.487	3.035	0.005
rs10596	16	31,488,916	G	C	8.450	3.034	0.005
rs11646054	16	31,495,671	C	G	8.445	3.035	0.005
rs9934336	16	31,495,873	A	G	-9.206	3.310	0.005
rs10685036	16	31,522,753	TTA	T	-8.531	3.091	0.006
rs11646911	16	31,483,622	T	C	8.356	3.033	0.006
rs11150626	16	31,491,982	C	T	-9.119	3.311	0.006
rs4889666	16	31,523,412	C	A	8.404	3.064	0.006
rs71375930	16	31,498,364	TCAAAA A	T	8.295	3.045	0.006
rs34081766	16	31,489,741	A	C	-8.982	3.311	0.007
rs13143	16	31,489,033	T	C	-8.974	3.311	0.007
rs13337037	16	31,478,711	A	G	-8.994	3.324	0.007
rs4889661	16	31,479,200	T	C	8.201	3.033	0.007
rs9926717	16	31,471,378	G	A	-8.932	3.312	0.007
rs8057401	16	31,524,685	T	C	-8.278	3.089	0.007
rs9935222	16	31,523,926	A	C	-8.276	3.090	0.007
rs11374860	16	31,475,477	TG	T	-8.802	3.330	0.008
rs8057207	16	31,524,580	T	C	-8.104	3.089	0.009
rs11150624	16	31,476,458	T	C	7.923	3.034	0.009
rs72790527	16	31,522,649	T	G	-9.987	3.973	0.012

Abbreviations: Chr=Chromosome, EFAL=Effect allele, NEFAL=Non-effect allele, SE=standard error.

Table S3. The association between SNPs and predicted 24-h urinary sodium excretion (mg/day) in the UK Biobank.

SNP	CHR	Locus	EFAL	NEFAL	Beta	SE	*P*
rs35884660	16	31,522,008	G	C	4.502	1.393	0.001
rs4889666	16	31,523,412	C	A	4.501	1.393	0.001
rs4889664	16	31,516,210	T	C	4.411	1.381	0.001
rs6565237	16	31,518,348	T	C	-4.350	1.365	0.001
rs111306768	16	31,526,948	CATGAA AGAAT	C	-4.263	1.363	0.002
16:31526674_CA_C	16	31,526,674	C	CA	-4.237	1.362	0.002
rs7192001	16	31,522,723	C	T	-4.221	1.358	0.002
rs8050548	16	31,523,801	A	G	-4.210	1.357	0.002
rs8057326	16	31,524,123	C	T	-4.202	1.357	0.002
rs7187900	16	31,523,113	G	A	-4.199	1.357	0.002
rs8062220	16	31,525,774	T	G	-4.196	1.357	0.002
rs8064191	16	31,525,871	G	A	-4.196	1.357	0.002
rs9935908	16	31,526,534	C	T	-4.194	1.357	0.002
rs8062603	16	31,525,960	A	G	-4.194	1.357	0.002
rs9922901	16	31,526,823	A	G	-4.190	1.357	0.002
16:31520375_GT_G	16	31,520,375	G	GT	-4.177	1.357	0.002
16:31525353_CCCTC CCTT_C	16	31,525,353	C	CCCTCC CTT	-4.136	1.359	0.002
rs71375930	16	31,498,364	TCAAAA A	T	4.197	1.382	0.002
rs35783178	16	31,520,759	CT	C	-4.156	1.373	0.002
rs4313820	16	31,480,318	G	C	4.069	1.377	0.003
rs10596	16	31,488,916	G	C	4.006	1.376	0.004
rs11646054	16	31,495,671	C	G	3.998	1.377	0.004
rs11646911	16	31,483,622	T	C	3.951	1.376	0.004
rs4889661	16	31,479,200	T	C	3.888	1.376	0.005
rs6565235	16	31,516,513	T	C	-4.211	1.520	0.006
rs8062314	16	31,516,690	A	C	-4.207	1.520	0.006
rs11150624	16	31,476,458	T	C	3.748	1.376	0.006
rs6565236	16	31,517,648	T	A	-4.135	1.520	0.007
rs11865835	16	31,509,816	C	T	-4.087	1.519	0.007
rs3934739	16	31,513,086	T	C	-4.045	1.519	0.008
rs9934336	16	31,495,873	A	G	-3.841	1.520	0.012

rs7188278	16	31,482,749	T	C	-3.835	1.520	0.012
rs11150626	16	31,491,982	C	T	-3.802	1.521	0.012
rs13337037	16	31,478,711	A	G	-3.806	1.527	0.013
rs13143	16	31,489,033	T	C	-3.768	1.521	0.013
rs34081766	16	31,489,741	A	C	-3.743	1.521	0.014
rs11374860	16	31,475,477	TG	T	-3.742	1.530	0.014
rs9926717	16	31,471,378	G	A	-3.696	1.521	0.015
rs10685036	16	31,522,753	TTA	T	-3.297	1.415	0.020
rs9935222	16	31,523,926	A	C	-3.161	1.414	0.025
rs8057401	16	31,524,685	T	C	-3.143	1.414	0.026
rs3116150	16	31,498,021	A	G	3.489	1.583	0.028
rs8057207	16	31,524,580	T	C	-3.067	1.414	0.030
rs72790527	16	31,522,649	T	G	-3.575	1.813	0.049

Abbreviations: Chr=Chromosome, EFAL=Effect allele, NEFAL=Non-effect allele, SE=standard error.

Table S4. Effects of SNPs on HbA1c and *SLC5A2* gene expression in the TransplantLines cohort.

SNP	Chr	Locus	HbA1c					Gene expression			
			EFAL	NEFAL	Beta	SE	*P*	EFAL	NEFAL	Estimate	*P*
rs45612043	16	31,484,598	C	A	-0.147	0.022	2.22E-11	C	A	-8.52	0.327
rs111510548	16	31,476,695	C	T	-0.075	0.015	7.58E-07	C	T	-2.85	0.515
rs11865835	16	31,509,816	C	T	-0.045	0.010	1.09E-05	C	T	-0.58	0.831
rs8062314	16	31,516,690	A	C	-0.045	0.010	1.11E-05	A	C	-0.58	0.831
rs6565235	16	31,516,513	T	C	-0.045	0.010	1.20E-05	T	C	-0.58	0.831
rs6565236	16	31,517,648	T	A	-0.044	0.010	1.23E-05	T	A	-0.54	0.842
rs9926717	16	31,471,378	G	A	-0.044	0.010	1.40E-05	G	A	-0.23	0.934
rs144413428	16	31,497,920	A	G	0.159	0.037	1.47E-05	A	G	10.08	0.265
rs13337037	16	31,478,711	A	G	-0.044	0.010	1.56E-05	A	G	-0.64	0.811
rs7188278	16	31,482,749	T	C	-0.044	0.010	1.56E-05	T	C	-0.54	0.841
rs3934739	16	31,513,086	T	C	-0.044	0.010	1.56E-05	T	C	-0.61	0.820
rs11150626	16	31,491,982	C	T	-0.043	0.010	2.06E-05	C	T	-0.54	0.841
rs9934336	16	31,495,873	A	G	-0.043	0.010	2.11E-05	A	G	-0.62	0.816
rs34081766	16	31,489,741	A	C	-0.043	0.010	2.27E-05	A	C	-0.54	0.841
rs13143	16	31,489,033	T	C	-0.043	0.010	2.27E-05	T	C	-0.54	0.841
rs9927250	16	31,506,900	G	A	-0.044	0.011	6.61E-05	G	A	-2.00	0.468
rs8057207	16	31,524,580	T	C	-0.037	0.010	8.26E-05	T	C	0.56	0.820
rs112853480	16	31,508,833	C	T	0.148	0.038	8.71E-05	C	T	9.46	0.340
rs8057401	16	31,524,685	T	C	-0.037	0.010	1.14E-04	T	C	0.59	0.811
rs9935222	16	31,523,926	A	C	-0.037	0.010	1.19E-04	A	C	0.53	0.829
rs3116150	16	31,498,021	A	G	0.041	0.011	1.24E-04	A	G	-2.20	0.454
rs4536493	16	31,503,751	G	A	-0.037	0.010	1.65E-04	G	A	-2.22	0.351
rs117800443	16	31,482,455	A	G	0.070	0.019	1.80E-04	A	G	3.68	0.545

Abbreviations: Chr=Chromosome, EFAL=Effect allele, NEFAL=Non-effect allele, SE=standard error.

Table S5. Effects of SNPs on HbA1c and *SLC5A2* gene expression in the NephQTL resource.

SNP	Chr	Locus	HbA1c					Gene Expression					
			EFAL	NEFAL	Beta	SE	*P*	EFAL	NEFAL	Beta	SE	*P*	Symbol
rs45612043	16	31,484,598	C	A	-0.147	0.022	2.22E-11	C	A	0.012	0.171	0.943	*SLC5A2*
rs9924771	16	31,495,577	G	A	-0.049	0.010	6.53E-07	G	A	-0.039	0.073	0.597	*SLC5A2*
rs111510548	16	31,476,695	C	T	-0.075	0.015	7.58E-07	C	T	0.100	0.124	0.423	*SLC5A2*
rs11374860	16	31,475,477	TG	T	-0.046	0.010	7.80E-06	TG	T	0.007	0.072	0.926	*SLC5A2*
rs11865835	16	31,509,816	C	T	-0.045	0.010	1.09E-05	C	T	0.021	0.070	0.766	*SLC5A2*
rs8062314	16	31,516,690	A	C	-0.045	0.010	1.11E-05	A	C	0.028	0.071	0.692	*SLC5A2*
rs6565235	16	31,516,513	T	C	-0.045	0.010	1.20E-05	T	C	0.021	0.070	0.766	*SLC5A2*
rs6565236	16	31,517,648	T	A	-0.044	0.010	1.23E-05	T	A	0.013	0.072	0.859	*SLC5A2*
rs9926717	16	31,471,378	G	A	-0.044	0.010	1.40E-05	G	A	0.012	0.069	0.862	*SLC5A2*
rs13337037	16	31,478,711	A	G	-0.044	0.010	1.56E-05	A	G	0.020	0.071	0.783	*SLC5A2*
rs7188278	16	31,482,749	T	C	-0.044	0.010	1.56E-05	T	C	0.021	0.070	0.764	*SLC5A2*
rs3934739	16	31,513,086	T	C	-0.044	0.010	1.56E-05	T	C	0.026	0.072	0.714	*SLC5A2*
rs11150626	16	31,491,982	C	T	-0.043	0.010	2.06E-05	C	T	0.005	0.071	0.948	*SLC5A2*
rs9934336	16	31,495,873	A	G	-0.043	0.010	2.11E-05	A	G	0.029	0.072	0.684	*SLC5A2*
rs34081766	16	31,489,741	A	C	-0.043	0.010	2.27E-05	A	C	0.021	0.072	0.770	*SLC5A2*
rs13143	16	31,489,033	T	C	-0.043	0.010	2.27E-05	T	C	0.018	0.072	0.798	*SLC5A2*
rs9927250	16	31,506,900	G	A	-0.044	0.011	6.61E-05	G	A	0.042	0.088	0.636	*SLC5A2*
rs8057207	16	31,524,580	T	C	-0.037	0.010	8.26E-05	T	C	0.068	0.069	0.324	*SLC5A2*
rs10685036	16	31,522,753	TTA	T	-0.037	0.010	1.10E-04	TTA	T	0.043	0.071	0.548	*SLC5A2*
rs8057401	16	31,524,685	T	C	-0.037	0.010	1.14E-04	T	C	0.047	0.069	0.496	*SLC5A2*
rs9935222	16	31,523,926	A	C	-0.037	0.010	1.19E-04	A	C	0.036	0.071	0.614	*SLC5A2*
rs3116150	16	31,498,021	A	G	0.041	0.011	1.24E-04	A	G	0.023	0.081	0.777	*SLC5A2*
rs4536493	16	31,503,751	G	A	-0.037	0.010	1.65E-04	G	A	0.029	0.074	0.701	*SLC5A2*
rs117800443	16	31,482,455	A	G	0.070	0.019	1.80E-04	A	G	-0.053	0.171	0.757	*SLC5A2*

Abbreviations: Chr=Chromosome, EFAL=Effect allele, NEFAL=Non-effect allele, SE=standard error.

Table S6. Effects of SNPs on HbA1c and *SLC5A2* gene expression in the eQTLGen consortium.

SNP	Chr	Locus	HbA1c					Expression (eQTL)				
			EFAL	NEFAL	Beta	SE	*P*	EFAL	NEFAL	Z-score	*P*	Symbol
rs45612043	16	31,484,598	C	A	-0.147	0.022	2.22E-11	C	A	-19.053	0.057	*SLC5A2*
rs9924771	16	31,495,577	G	A	-0.049	0.010	6.53E-07	A	G	-22.985	0.022	*SLC5A2*
rs111510548	16	31,476,695	C	T	-0.075	0.015	7.58E-07	C	T	5.199	2.00E-07	*SLC5A2*
rs11865835	16	31,509,816	C	T	-0.045	0.010	1.09E-05	C	T	5.736	9.69E-09	*SLC5A2*
rs8062314	16	31,516,690	A	C	-0.045	0.010	1.11E-05	A	C	5.727	1.02E-08	*SLC5A2*
rs6565235	16	31,516,513	T	C	-0.045	0.010	1.20E-05	T	C	5.746	9.15E-09	*SLC5A2*
rs6565236	16	31,517,648	T	A	-0.044	0.010	1.23E-05	T	A	6.112	9.86E-10	*SLC5A2*
rs9926717	16	31,471,378	G	A	-0.044	0.010	1.40E-05	G	A	5.481	4.22E-08	*SLC5A2*
rs144413428	16	31,497,920	A	G	0.159	0.037	1.47E-05	A	G	-0.784	0.433	*SLC5A2*
rs13337037	16	31,478,711	A	G	-0.044	0.010	1.56E-05	A	G	5.912	3.39E-09	*SLC5A2*
rs7188278	16	31,482,749	T	C	-0.044	0.010	1.56E-05	T	C	5.478	4.31E-08	*SLC5A2*
rs3934739	16	31,513,086	T	C	-0.044	0.010	1.56E-05	T	C	6.073	1.25E-09	*SLC5A2*
rs11150626	16	31,491,982	C	T	-0.043	0.010	2.06E-05	C	T	5.980	2.23E-09	*SLC5A2*
rs9934336	16	31,495,873	A	G	-0.043	0.010	2.11E-05	A	G	5.959	2.54E-09	*SLC5A2*
rs34081766	16	31,489,741	A	C	-0.043	0.010	2.27E-05	A	C	5.968	2.40E-09	*SLC5A2*
rs13143	16	31,489,033	T	C	-0.043	0.010	2.27E-05	T	C	6.060	1.36E-09	*SLC5A2*
rs9927250	16	31,506,900	G	A	-0.044	0.011	6.61E-05	A	G	-2.083	0.037	*SLC5A2*
rs8057207	16	31,524,580	T	C	-0.037	0.010	8.26E-05	T	C	5.090	3.58E-07	*SLC5A2*
rs112853480	16	31,508,833	C	T	0.148	0.038	8.71E-05	C	T	0.218	0.828	*SLC5A2*
rs8057401	16	31,524,685	T	C	-0.037	0.010	1.14E-04	T	C	4.995	5.88E-07	*SLC5A2*
rs9935222	16	31,523,926	A	C	-0.037	0.010	1.19E-04	A	C	5.102	3.36E-07	*SLC5A2*
rs3116150	16	31,498,021	A	G	0.041	0.011	1.24E-04	A	G	-13.166	0.188	*SLC5A2*
rs4536493	16	31,503,751	G	A	-0.037	0.010	1.65E-04	A	G	-3.006	0,003	*SLC5A2*
rs117800443	16	31,482,455	A	G	0.070	0.019	1.80E-04	A	G	-17.393	0.082	*SLC5A2*

Abbreviations: Chr=Chromosome, EFAL=Effect allele, NEFAL=Non-effect allele, SE=standard error.

Table S7. CADD score for SNPs significantly associated with HbA1c in UK Biobank.

SNP	Chr	Locus	Alternative allele	Reference allele	Symbol	CADD PHRED scaled score
rs9926717	16	31,471,378	G	A	*ARMC5*	8.528
rs11374860	16	31,475,477	G	T	*ARMC5*	0.020
rs111510548	16	31,476,695	C	T	*ARMC5*	4.538
rs13337037	16	31,478,711	A	G	*ARMC5/TGFB1I1*	8.756
rs117800443	16	31,482,455	A	G	*ARMC5/TGFB1I1*	3.153
rs7188278	16	31,482,749	T	C	*ARMC5/TGFB1I1*	10.84
rs45612043	16	31,484,598	C	A	*TGFB1I1*	12.79
rs13143	16	31,489,033	T	C	*TGFB1I1*	6.091
rs34081766	16	31,489,741	A	C	*SLC5A2/TGFB1I1*	0.228
rs1251169601	16	31,490,046	A	C	*SLC5A2/TGFB1I1*	0.954
rs11150626	16	31,491,982	C	T	*SLC5A2/TGFB1I1*	5.413
rs9924771	16	31,495,577	G	A	*SLC5A2*	1.858
rs9934336	16	31,495,873	A	G	*SLC5A2/C16orf58*	2.596
rs144413428	16	31,497,920	A	G	*SLC5A2/C16orf58*	3.927
rs3116150	16	31,498,021	A	G	*SLC5A2/C16orf58*	4.732
rs4536493	16	31,503,751	G	A	*SLC5A2/C16orf58*	5.674
rs9927250	16	31,506,900	G	A	*SLC5A2/C16orf58*	6.287
rs112853480	16	31,508,833	C	T	*C16orf58*	3.369
rs11865835	16	31,509,816	C	T	*C16orf58*	0.273
rs3934739	16	31,513,086	T	C	*C16orf58*	2.832
rs6565235	16	31,516,513	T	C	*C16orf58*	1.866
rs8062314	16	31,516,690	A	C	*C16orf58*	3.146
rs6565236	16	31,517,648	T	A	*C16orf58*	0.225
rs10685036	16	31,522,753	A	T	*C16orf58*	7.636
rs9935222	16	31,523,926	A	C	*C16orf58*	1.258
rs8057207	16	31,524,580	T	C	*C16orf58*	0.034
rs8057401	16	31,524,685	T	C	*C16orf58*	5.330

Abbreviations: Chr=Chromosome.

Figure S1. ***SLC5A2*** **gene expression in various tissues.**

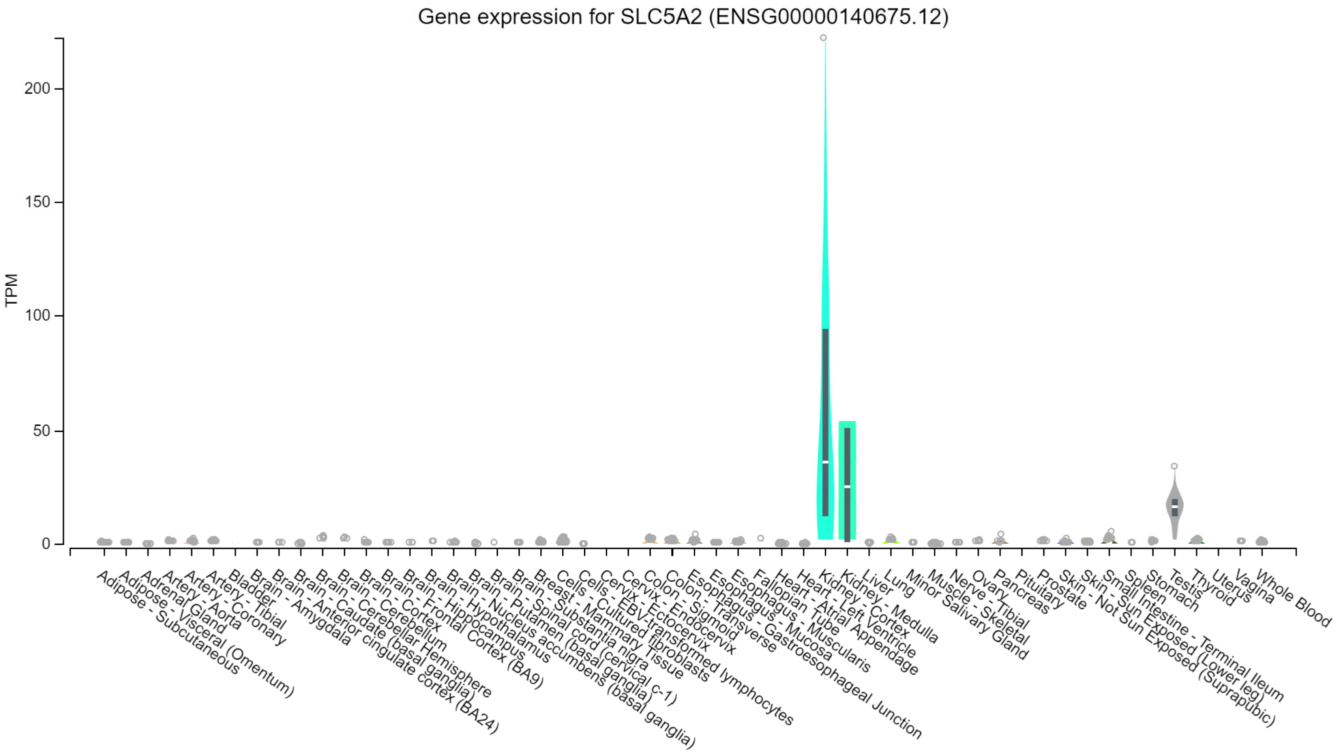

SLC5A2 gene expression in different tissue types. The graph was generated by the Genotype-Tissue Expression (GTEx) Portal on 14/04/2020. (TPM=transcripts per million)

Figure S2. Genetic correlation of the 25 SNPs which significantly associated with HbA1c in UK Biobank

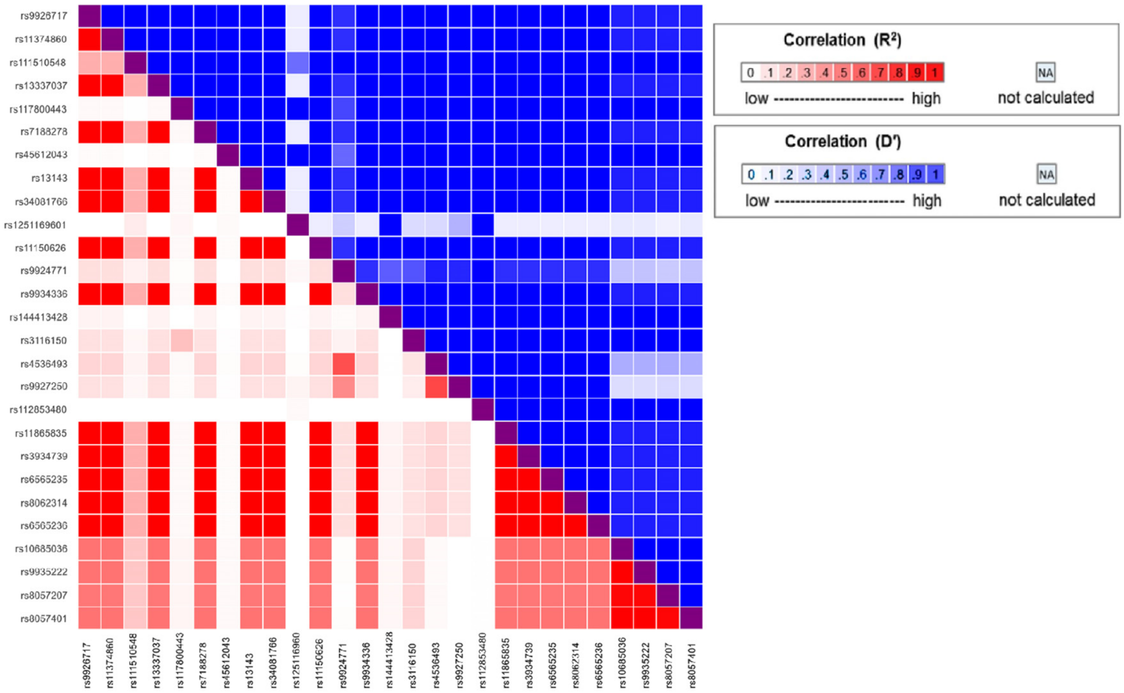

Shown are the pairwise linkage disequilibrium statistics results (r^2 in red and D' in blue). This interactive heatmap matrix was generated by using LDmatrix Tool of LDlink.
